# Apigenin-7-O-glucoside versus apigenin: Insight into the modes of anticandidal and cytotoxic actions

**DOI:** 10.17179/excli2017-300

**Published:** 2017-05-23

**Authors:** Marija Smiljkovic, Danijela Stanisavljevic, Dejan Stojkovic, Isidora Petrovic, Jelena Marjanovic Vicentic, Jelena Popovic, Simona Golic Grdadolnik, Dejan Markovic, Snežana Sankovic-Babice, Jasmina Glamoclija, Milena Stevanovic, Marina Sokovic

**Affiliations:** 1 Department of Plant Physiology, Institute for Biological Research "Siniša Stankovic", University of Belgrade, Bulevar Despota Stefana 142, 11000 Belgrade, Serbia; 2 Institute of Molecular Genetics and Genetic Engineering, University of Belgrade, Vojvode Stepe 444a, PO Box 23, 11010 Belgrade, Serbia; 3 Laboratory of Biomolecular Structure, National Institute of Chemistry, Hajdrihova 19, 1000 Ljubljana, Slovenia; 4 Clinic for Pediatric and Preventative Dentistry, Faculty of Dental Medicine, Rankeova 4, Belgrade; 5 ENT Clinic, Clinical Hospital Centre Zvezdara, Presevska 31, 11000 Belgrade, Serbia

**Keywords:** apigenin-7-O-glucoside, apigenin, antifungal, Candida spp., cytotoxic, HCT116

## Abstract

Bioactive potential of apigenin derivative apigenin-7-*O*-glucoside related to its antifungal activity on *Candida* spp. and cytotoxic effect on colon cancer cells was studied and compared with bioactive potential of apigenin. Antifungal activity was tested on 14 different isolates of *Candida* spp. using membrane permeability assay, measuring inhibition of reactive oxidative species and inhibition of CYP51 *C. albicans* enzyme. Cytotoxic potential of apigenin-7-*O*-glucoside was tested on colon cancer HCT116 cells by measuring cell viability, apoptosis rate and apoptosis- and colon cancer-related gene expression. Obtained results indicated considerable antifungal activity of apigenin-7-*O*-glucoside towards all *Candida* isolates. Breakdown of *C. albicans *plasma membrane was achieved upon treatment with apigenin-7-*O*-glucoside for shorter period of time then with apigenin. Reduction of intra- and extracellular reactive oxidative species was achieved with minimum inhibitory concentrations of both compounds, suggesting that reactive oxidative species inhibition could be a mechanism of antifungal action. None of the compounds exhibited binding affinity to *C. albicans* CYP51 protein. Besides, apigenin-7-*O*-glucoside was more effective compared to apigenin in reduction of cell's viability and induction of cell death of HCT116 cells. Treatment with both compounds resulted in chromatin condensation, apoptotic bodies formation and apoptotic genes expression in HCT116 cells, but the apigenin-7-*O*-glucoside required a lower concentration to achieve the same effect. Compounds apigenin-7-*O*-glucoside and apigenin displayed prominent antifungal potential and cytotoxic effect on HCT116 cells. However, our results showed that apigenin-7-*O*-glucoside has more potent activity compared to apigenin in all assays that we used.

## Introduction

There is a constant need for search of novel antifungal drugs considering the fact that resistance and multi-resistance occurs very often to synthetic drugs currently in use for treatment of fungal infections. In the field of naturally occurring substances that might have a potential antifungal activity, there are many opportunities for research. One of the major groups with highly potent bioactive compounds is a group of flavonoids which is promising regarding discovering new antifungal compounds and compounds capable to reduce the incidence of different cancer types (Wesołowska, 2011[35]; Kandaswami et al., 2005[10]).

Literature data suggest that there is a link between pathogen fungus present in intestinal mycobiota and the incidence of adenomas. In particular, Luan et al. (2015[19]) showed that the presence of pathogen fungus in intestine may be common among patients with adenomas. *Candida* was one of the genera with relatively high abundance present in adenomas (7 %) and adjacent tissue samples (1 %). More than 80 % of sporadic colorectal cancer (CRC) cases were induced by colorectal adenoma (Ullman and Itzkowitz, 2011[32]). This type of cancer is the third most common cancer in males and the second most common cancer in females worldwide; over 1.2 million CRC diagnoses and 608,700 CRC deaths were recorded in 2008 (Jemal et al., 2010[7]). Advanced adenomas can further develop into carcinoma. Although, during the past decade, colorectal screening leads to decreased incidence and mortality (Jorgensen and Knudtson, 2015[8]), there is a constant demand for development of novel drugs and identification of natural compounds with antitumor activity. 

Apigenin-7-*O*-glucoside (AP7Glu) is a stable natural flavonoid, with better solubility compared to other flavonoids such as apigenin. They both have similar anti-inflammatory capacity (Kowalski et al., 2005[13]). AP7Glu has multiple biological activities and is currently prescribed to treat inflammatory diseases such as upper respiratory infections (Bhaskaran et al., 2010[1]). It was recently shown that AP7Glu possessed anxiolytic potential in rats, comparable to the reference drugs apigenin and diazepam (Kumar and Bhat, 2012[14]). Apigenin is a non-toxic and non-mutagenic flavone subclass of flavonoids, present in fruits and vegetables (cardoon, celery, artichoke, parsley etc.), some of which are widely marketed as dietary and herbal supplements (Sharma et al., 2014[29]). Apigenin has received considerable attention due to its significant anticancer, antiviral, antibacterial, antioxidant, pro-apoptotic and anti-inflammatory effects (Kanazawa et al., 2006[9]; Cai et al., 2011[3]).

The aim of this study was to bring new insight into bioactive potential of apigenin derivative apigenin-7-*O*-glucoside, related to its antifungal activity and cytotoxic effect on colon cancer cells and to compare it with bioactive potential of apigenin. Beside their comparison, our intent was to get better insight of potential dual effect of tested bioactive compounds necessary for different aspects of colon cancer treatment.

## Material and Methods

### Apigenin-7-O-glucoside and apigenin

Flavonoid compounds AP7Glu and apigenin were commercially available (Extrasynthese, France).

### Anti-candidal activity

#### Microbial culture conditions

Eleven strains of *C. albicans* were used in the experiments, including isolates of *C. krusei*, *C. glabrata* and *C. tropicalis*. Nine of the strains used were clinical isolates and two were reference strains *Candida albicans* ATCC 10231 and *Candida tropicalis* ATCC 750. All clinical isolates were obtained by rubbing a sterile cotton swab over oral mucosa from patients at the Department of Pediatric and Preventive Dentistry, Faculty of Dental Medicine, University of Belgrade, Serbia, upon obtaining informed written consent. Strains of *Candida* spp. were maintained on Sabourand Dextrose Agar (Merck, Germany) at 4 °C and subcultured once a month. Identification of *Candida* spp. was done using biochemical profiling with API 20C and with CHROMagar plates. 

#### Microdilution method

Minimum inhibitory (MIC) and minimum fungicidal (MFC) concentrations were determined by microdilution method in 96 well microtitre plates, described by Douk et al. (1995[5]) and EUCAST (2002[6]). As a positive control commercial mycotic drug ketoconazole (Sigma-Aldrich, St. Louis, MO) was used.

### Insights in the mode of antimicrobial action of AP7Glu and apigenin

#### Nucleotide leakage - membrane permeability assay

The effect of apigenin-7-*O*-glucoside on membrane permeability (nucleotide leakage) was evaluated according to Tang et al. (2008[31]) with some modifications and compared to effect of apigenin. The culture of *C. albicans* 475/15 incubated overnight at 37 °C was washed and resuspended in 10 mM PBS (pH 7.4), reaching the final density of 10^8 ^CFU/ ml. Strain was incubated with the target molecules at the 1½ MICs for different time intervals: 0, 15, 30, 45 and 60 min; *C. albicans* incubated with 10 mM PBS (pH 7.4) was used as control. The mixture was filtered through 0.22 μm pore size filter to remove the yeast cells. The optical density of the filtrate was measured at 260 nm and 280 nm with Agilent/HP 8453 UV-Visible Spectrophotometer Agilent Technologies, USA) at room temperature (25 °C).

#### Determination of extracellular and intracellular ROS in C. albicans

These studies were carried out with suspensions of *C. albicans* 475/15, supplemented with MICs, ½ MICs and ¼ MICs of apigenin-7-*O*-glucoside and apigenin. For the nitro blue tetrazolium (NBT) reaction (Páez et al., 2010[24]) 0.4 mL of yeast suspension treated overnight with apigenin-7-*O*-glucoside and apigenin (OD600 nm 0.8) and 0.5 mL of 1 mg/mL NBT were incubated for 30 min at 37 °C. Then, 0.1 mL of 0.1 M HCl was added and the tubes were centrifuged at 2500 g for 10 min, with the blue color of supernatants being measured at 575 nm (ROS extracellular). The separated pellets were treated with 0.6 mL dimethyl sulfoxide (DMSO) to extract the reduced NBT, and finally, 0.8 mL phosphate saline buffer was added and OD575 nm was determined (ROS intracellular) using Agilent/HP 8453 UV-Visible Spectrophotometer (Agilent Technologies, USA).

#### Investigation of binding properties of apigenin-7-O-glucoside and apigenin for CaCYP51 enzyme

Sterol 14α-demethylase (CYP51) was previously isolated and kindly provided by Laboratory of Biomolecular Structure at National Institute of Chemistry, Ljubljana, Slovenia. Binding properties were investigated using UV-Visible spectroscopy. Different concentration of investigated compounds (0, 2, 8, 16, 32, 64, 128, 256, 300 µM) were mixed with CYP51 protein from *Candida albicans*. Spectra were recorded from 350 to 500 nm, and possible ligand-induced spectral changes were monitored as difference type II spectral responses (Zelenko et al., 2014[37]). 

### Cytotoxic activity

#### Cell culture

Human HCT116 (ATCC-CCL-227) colon cancer cells were grown in Dulbecco's Modified Eagle's medium (DMEM) supplemented with 10 % fetal bovine serum (FBS), 4500 mg/L glucose, 2 mmol/L L-glutamine and penicillin/streptomycin (all from Invitrogen™, USA). The cells were maintained at 37 °C in 5 % CO_2_.

#### MTT assay

HCT116 cells were seeded overnight at a density of 3 x 10^4 ^cells per well in 96 well plate. After 24 h, cells were treated with vehicle DMSO and various concentrations of apigenin or apigenin-7-*O*-glucoside for 48 h. After incubation, the cell's viability was determined by adding MTT (3-[4, 5-dimethylthiazol-2-yl]-2,5-diphenyltetrazolium bromide) at final concentration 5 mg/ml (Merck, Germany). MTT containing medium was aspirated after 2 h and DMSO was added to each well to achieve solubilization of the formazan crystal. The absorbance at 550 nm was recorded using plate reader Infinite 200 pro (Tecan, Austria).

### Insights in the mode of cytotoxic action of AP7Glu and apigenin

#### DAPI staining of nuclear morphological changes of HCT116 cells

HCT116 cells were seeded on cover slips overnight at a density of 9 x 10^4^ cells per well in 12 well plate. After 24 h, cells were treated with calculated IC_50_ for AP7Glu and apigenin, or with DMSO as a control. 48 h after treatment cells were fixed in 4 % paraformaldehyde (PFA) for 20 min at room temperature. After fixation, nuclei were stained with 0.1 mg/ml diamino phenylindole-DAPI (Sigma-Aldrich, USA). Cells were visualized by OLYMPUS BX41 fluorescence microscope (Applied Imaging Corporation, USA) using the fluorescence filter 330-380 nm, captured with 60 x magnificence. Cells were counted at least in four different fields with a total number of 200 cells. The percentage of apoptotic cells (apoptotic bodies) was calculated as the ratio of apoptotic cells to total cells counted.

#### Apoptosis assay using a double staining method with Annexin V-FITC/PI

Apoptosis assays were conducted using the APOPTEST™-FITC kit (Dako, Agilent Technologies, USA) according to the manufacturer's instructions. The cells were treated with IC_50_ concentration for AP7Glu or apigenin for 48 hours or with DMSO. The cells were washed twice with cold PBS, resuspended in 1× Binding Buffer at a final number of 1 × 10^6^ cells/ml and 5 μl Annexin V and propidium iodide (PI) were added. The cells were gently mixed, incubated for 10 min in the dark at room temperature, and analyzed by Partec CyFlow® Space (Partec GmbH, Germany). The flow cytometer collected 100.000 events and analysis was performed using Flomax 2.9 software. 

### RT-PCR analysis

Total RNA was isolated using TRI-Reagent (Ambion®, Invitrogen, USA) according to the manufacturer's instructions. RNA was treated with DNase I using a DNA-Free™ kit (Ambion, Invitrogen, USA) and subjected to cDNA synthesis. Total RNA (1 μg) was reverse transcribed using High Capacity cDNA Reverse Transcription Kit (Applied Biosystems, USA) according to the manufacturer's protocol. 

For quantitative PCR analysis, cDNAs were subjected to real time PCR using Power SYBR Green PCR Master Mix (Applied Biosystems, USA) in 7500 Real Time PCR Systems (Applied Biosystems, USA).The synthesized cDNAs were used as templates for amplification with primers specific for *SOX9*, *p53*, *c-Myc*, *Cyclin D1*, *Bax* and *GAPDH*. 

Primers for *p53 *amplification were as follows:

5'-CCCCTCCTGGCCCCTGTCATCTTC-3' (forward) and 5'-GCAGCGCCTCACAACCTCCGTCAT 3' (reverse). *C-Myc* was amplified using primers 5' CAAGAGGCGAACACACAACGTC3' (forward) and 5' CTGTTCTCGTCGTTTCCGCAAC 3' (reverse). For *Cyclin D1* amplification we have used 5'-CCTGTCCTACTACCGCCTCA-3' (forward) and 5'-TCCTCCTCTTCCTCCTCCTC-3' (reverse) primers, for *Bax* amplification we have used 5'-TGGCAGCTGACATGTTTTCTGAC-3' (forward) and 5'-TCACCCAACCACCCTGGTCTT-3' (reverse), for *SOX9* amplification we have used5'- CTTCTGAACGAGAGCGAGA-3' (forward) and 5'-CTGCCCGTTCTTCACCGACTTC-3' (reverse) primers. *GAPDH* was amplified with 5′-GGACCTGACCTGCCGTCTAG-3′ (forward) and 5′-CCACCACCCTGTTGCTGTAG-3′ 

(reverse) to control for equivalent amounts of cDNA per reaction. All samples were measured in triplicate and the mean value was considered. The relative level of analyzed gene's expression was determined using a comparative quantification algorithm where the resulting ΔΔCt value was incorporated to determine the fold difference in expression (2^−ΔΔCt^). Relative mRNA level was presented as a percentage of mRNA expression in control cells treated with DMSO.

### Statistical analyses

Statistical analyses were performed with SPSS statistical software (version 20). The data represents means ± SEM from three independent experiments. Statistical analyses were performed by Student's *t*-test and p value ≤ 0.05 was considered significant. 

## Results

### AP7Glu exhibits increased anti-candidal activity compare to apigenin

Results of the anti-candidal activity of AP7Glu (0.05-0.2 mg/mL), apigenin (0.1-0.3 mg/mL) and commercial drug ketoconazole (0.0016-0.1 mg/mL) are presented in Table 1(Tab. 1). Obtained results showed that all tested strains were more sensitive to AP7Glu than to apigenin. In particular, range of MICs and MFCs was 0.05 - 0.10 mg/mL for AP7Glu, while treatment with apigenin resulted with MIC 0.10 mg/mL and MFC 0.20 mg/mL. The most resistant strain to both compounds was *C. krusei* with the same values of MICs and MFCs (0.15 and 0.30 mg/mL, respectively). Positive control ketoconazole was used for monitoring anti-candidal activity and all species were more sensitive to commercial drug when compared to tested compounds. These results propose that AP7Glu has more potent anti-candidal activity compared to apigenin. 

### AP7Glu interferes with membrane integrity of C. albicans more rapidly than apigenin

**Figure 1:**
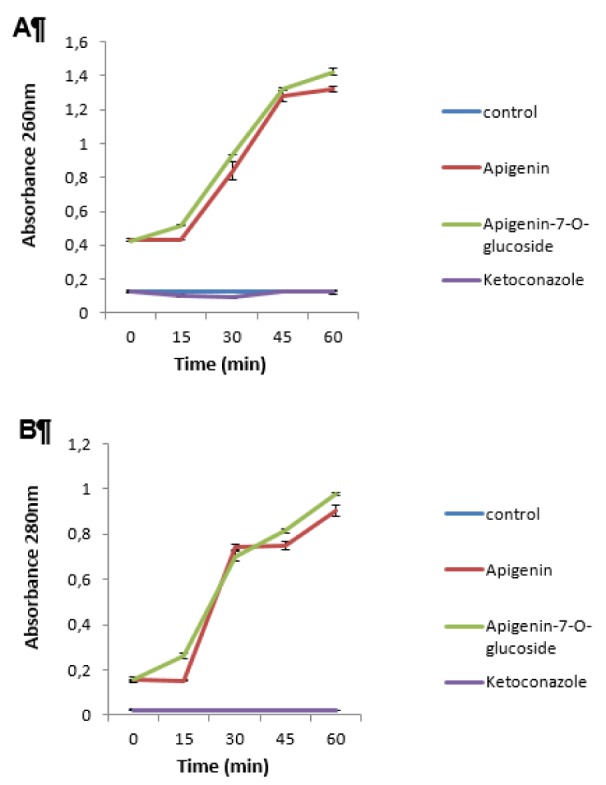
Nucleotide leakage in *Candida albicans* cells during treatment with apigenin, apigenin-7-*O*-glucoside and ketoconazole at 1½ MIC concentrations (0.15 mg/mL, 0.075 mg/mL and 0.00465 mg/mL, respectively) reported with absorbance on 260nm (A) and 280nm (B), untreated *C. albicans* cells were used as control.

In order to evaluate the breakdown of plasma membrane in the presence of effective concentration of tested compounds, a membrane permeability assay was performed. Total nucleotide leakage from cells of *C. albicans *was observed as a function of incubation time with AP7Glu and apigenin. At optical density at 260 and 280 nm, treatment with AP7Glu increased absorbance more profoundly than treatment with apigenin (Figure 1(Fig. 1)). Ketoconazole did not induce membrane breakdown at tested concentration. Absorbance of the control samples was not changed during the time. 

Obtained results for nucleotide leakage are a good indicator of compromised membrane integrity which implies that both apigenin and AP7Glu might directly act on the cell membrane surface inducing its rupture and release of intracellular genetic material. However, AP7Glu demonstrated more disturbing effect on *C. albicans* plasma membrane compared to apigenin.

### AP7Glu has higher potential to inhibit extra- and intracellular ROS production of C. albicans compared to apigenin

**Figure 2:**
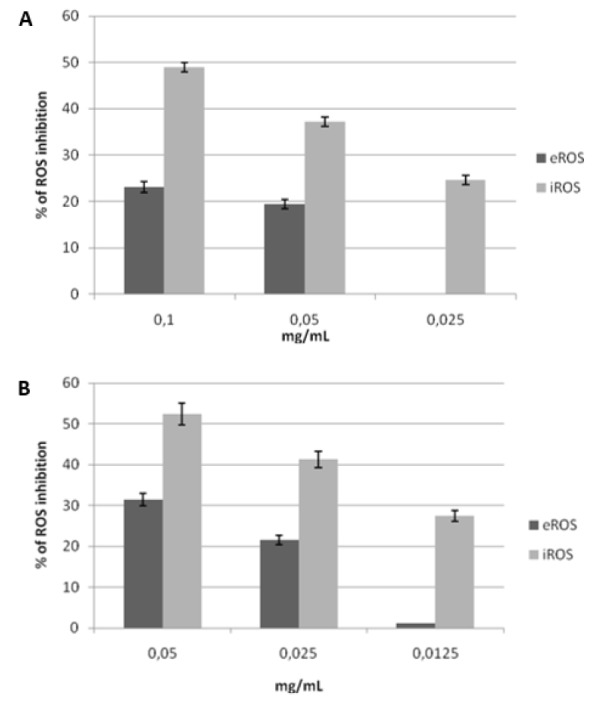
Percentage of extracellular (eROS) and intracellular (iROS) inhibition of reactive oxygen species in *C. albicans* cells treated with apigenin (A) and apigenin-7-*O*-glucoside (B) at MIC, ½ MIC and ¼ MIC concentrations. Ketoconazole did not cause any changes in ROS levels, data not shown.

*C. albicans* is capable of generating significant amounts of ROS which is in correlation with its ability to invade host tissue by provoking oxidative damage in host cells at MIC and subMIC concentrations (Schröter et al., 2000[26]), ROS can react with polyunsaturated fatty acids in cellular membranes, sulfhydryl bonds in proteins and nucleotides, and therefore induce tissue injury in yeast infections (Machlin and Bendich, 1987[20]; Nishikawa et al., 1997[23]). We analyzed intracellular and extracellular ROS production by *C. albicans* in the presence of AP7Glu, apigenin and commercial antifungal drug ketoconazole. The results indicated decreasing amounts of extracellular and intracellular ROS in the presence of AP7Glu and apigenin which was shown to be dose-dependent (Figure 2A, B(Fig. 2)). The similar pattern was noted for extracellular ROS, except that intracellular ROS was inhibited more profoundly than extracellular ROS with both compounds at MIC values. Treatment with ketoconazole did not cause changes neither in extracellular nor in intracellular ROS levels. AP7Glu and apigenin possessed similar activity, highlighting AP7Glu as more potent ROS inhibitor at lower concentration.

### AP7Glu and apigenin do not show binding affinities to candidal CYP51 protein

CYP51 is enzyme essential for *Candida albicans* involved in ergosterol biosynthetic pathway, and it's main target for azole antifungal drugs (Kelly et al., 2003[12]; Warrilow et al., 2010[34]). In this study we found that none of the tested compounds bound to CYP51 protein from *C. albicans *(data not shown), while ketoconazole has mode of action which involves CYP51 inhibition since it bounds candidal enzyme with Kd CaCYP51 < 0.05 µM, but it also showed affinity towards human protein with Kd hCYP51 < 0.05 µM. This result indicated different mechanism of anticandidal activity of AP7Glu and apigenin in comparison to available antifungal azole drugs and draws attention to the unselective binding affinities of ketoconazole.

### AP7Glu reduce viability of colon cancer cells more than apigenin

**Figure 3:**
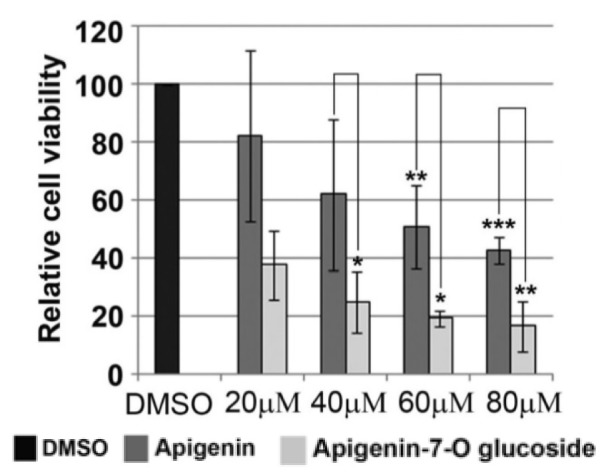
Figure 3: The effect of apigenin and AP7Glu on HCT116 cell's viability. Increasing amounts (20-80 μM) of apigenin and AP7Glu were used for treatment of HCT116 cells. 48 h after treatment cell viability was determined by MTT assay. Relative cell's viability for cells treated with apigenin was calculated as a percentage of HCT116 cells viability treated with DMSO that was set as 100 %. Relative cell's viability for cells treated with AP7Glu was calculated compared to apigenin. Results were presented as the means ± SEM of at least three independent experiments. P values were calculated using Student's *t*-test, *p ≤ 0.05, **p ≤ 0.01, ***p ≤ 0.001.

The effect of AP7Glu on the HCT116 colon cancer cell's viability was examined by the MTT assay and compared to the effect of apigenin. Cells were treated with various doses of AP7Glu or apigenin and then cell's viability were tested 48 h after treatment. As shown in Figure 3(Fig. 3), both AP7Glu and apigenin led to reduction in cell's viability in a dose-dependent manner. Moreover, the cytotoxic effect of AP7Glu was approximately 4-fold stronger compared to apigenin. In particular, determined IC_50_ values for apigenin and AP7Glu were 62 and 15 µM, respectively. 

### AP7Glu induces changes in nuclear morphology of HCT116 cells 

**Figure 4:**
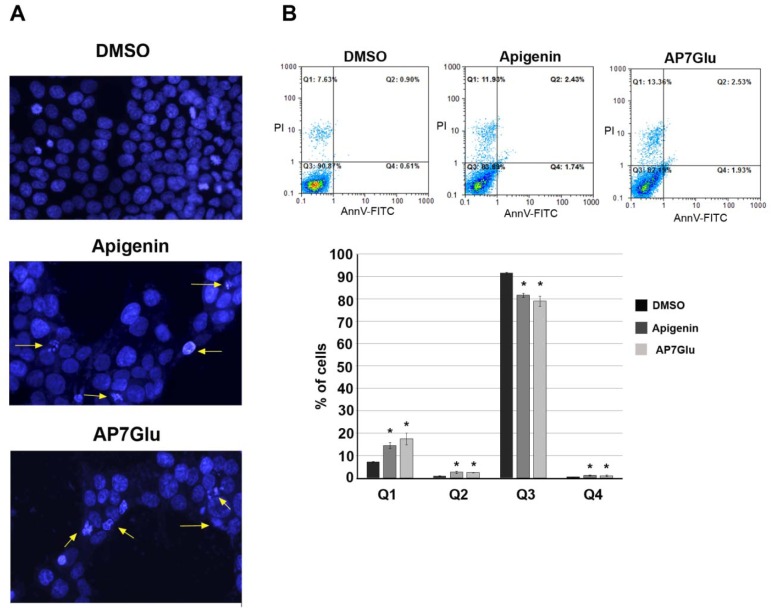
AP7Glu induces cell death of HCT116 cells. A) Representative composite images showing morphological changes of HCT116 cells detected with DAPI staining. Cells were treated with IC_50_ concentrations of apigenin or AP7Glu for 48 h, and imaged by fluorescence microscope. Apoptotic bodies formation are marked by yellow arrows. B) Flow cytometry analysis of Annexin-FITC staining and propidium iodide accumulation after treatment of HCT116 cells with apigenin or AP7Glu. Cells were treated either with DMSO or corresponding IC_50_ concetrations (treatment) for 48 h. One representative analysis was presented in upper panel. Results of quantitative analyses of PI and Annexin positive cells were presented as the means ± SEM of at least three independent experiments. P values were calculated using Student's *t*-test, *p ≤ 0.05. Q1: PI+cells; Q2: PI+/Annexin+cells; Q3: PI-/Annexin-cells; Q4: Annexin+cells.

Apoptosis is characterized by morphological alterations of nuclei, like condensation of nuclear chromatin and fragmentation of residual nuclear structures into apoptotic bodies (Lazebnik et al., 1993[15]). DAPI is a dye known to form fluorescent complexes with natural double-stranded DNA, showing fluorescence specificity for AT, AU and IC clusters (Kapuscinski and Yanagj, 1979[11]). Therefore, DAPI is a good tool for visualization of chromatin condensation with fluorescent microscopy (Ziegler and Groscurth, 2004[38]). As shown in Figure 4A(Fig. 4), control group of HCT116 cells nuclei were round in shape and stained homogeneously with DAPI. In contrast, after 48 h treatment of HCT116 cells with AP7Glu we have detected formation of apoptotic bodies with characteristic nuclear morphological changes (Figure 4A(Fig. 4), yellow arrows). The same effect was detected after treatment with apigenin (Figure 4A(Fig. 4), yellow arrows). In particular, in DMSO treated cells we have detected approximately 0,7 % of cells undergoing apoptosis (apoptotic bodies), whereas in apigenin and AP7Glu treated cells we have detected 12,5 % and 10,5 % of apoptotic bodies, respectively. Upon treatment, there was visible decrease in cell number that correlates with results of MTT assay. Furthermore, mitotic cells were not visible (Figure 4A(Fig. 4)). These results suggested that treatment of HCT116 cells with AP7Glu, as well as with apigenin led to induction of cell death.

### Treatment of HCT116 cells with AP7Glu promote cell death

HCT116 cells were treated with IC_50_ concentrations of apigenin or AP7Glu for 48 h and the proportion of apoptosis and necrosis was analyzed using Annexin V/propidium iodide staining. Obtained results suggested that treatment with AP7Glu led to minor induction of apoptosis (approximately 1 % of cells detected in early apoptosis and 2.5 % of cells in late apoptosis) (Figure 4B(Fig. 4)) while 17.5 % of cells underwent necrosis (Figure 4B(Fig. 4)). Treatment of HCT116 cells with apigenin led to the same effect, where approximately 1 % of cells was detected in early apoptosis, 2.5 % of cells in late apoptosis and approximately 14.5 % of cells underwent necrosis (Figure 4B(Fig. 4)). Therefore, by flow cytometry we confirmed that AP7Glu is able to induce cell death. It is important to point out that AP7Glu displayed the same effect in induction of cell death compared to apigenin, but approximately at 4-fold lower concentration. 

### AP7Glu affects apoptosis-associated gene expression in HCT166 cells

**Figure 5:**
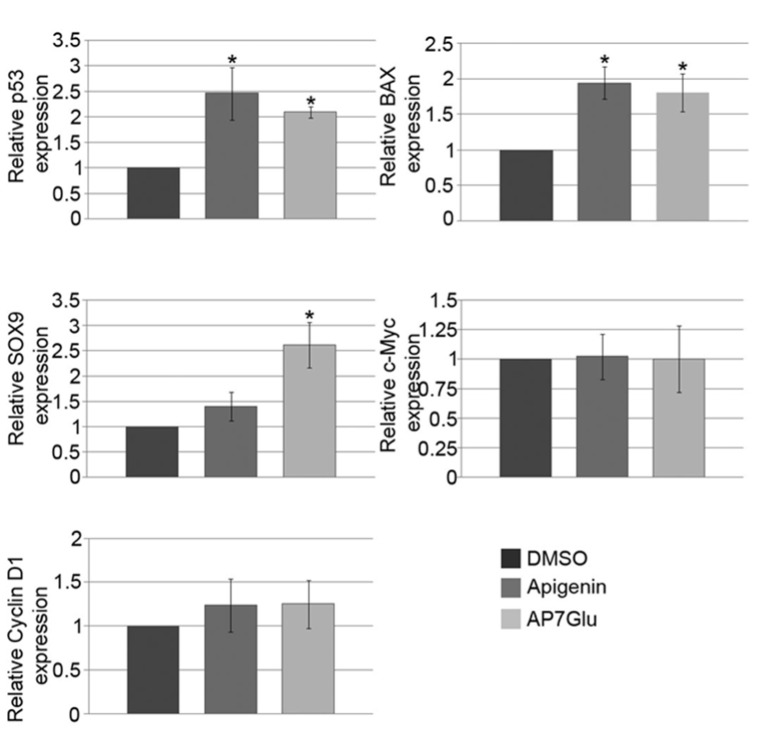
The effect of apigenin and AP7Glu treatment of HCT116 cells on the expression level of *p53*, *Bax*, *c-Myc*, *Cyclin* *D1* and *SOX9*. Cells were treated with IC_50_ concentrations of apigenin or AP7Glu for 48 h and the level of genes expression was quantified by qRT-PCR. Relative gene expression was presented as percentage of expression in cells treated with DMSO that was set as 100 %. Results were presented as the means ± SEM of at least three independent experiments performed in triplicates. P values were calculated using Student's *t*-test. *p ≤ 0.05

In order to investigate whether this cytotoxic effect of AP7Glu leads to changes in expression of molecular markers involved in regulation of cell cycle and apoptosis, we investigated the expression of *p53*, *Bax* and *Cyclin D1* upon treatment with IC_50_ concentration of AP7Glu. Furthermore, we analyzed the expression of two transcription factors, *c-Myc* and *SOX9*, which expression was shown to be deregulated in colon cancer (Chen et al., 2007[4]; Lü et al., 2008[17]; Matheu et al., 2012[22]). Presented results show that treatment of HCT116 cells with AP7Glu led to approximately 1.8-fold induction in *Bax* expression, 2-fold induction in *p53* expression, while no significant changes were observed regarding *Cyclin D1* and *c-Myc* gene expression (Figure 5(Fig. 5)). In parallel, we investigated the effect of apigenin on the expression level of the same genes. Similarly to AP7Glu, treatment of HCT116 cells with apigenin led to approximately 2-fold induction in *Bax* expression, 2.5-fold induction in *p53* expression, while no significant changes were observed regarding *Cyclin D1*, *c-Myc* (Figure 5(Fig. 5)). Regarding *SOX9* gene expression upon treatment with AP7Glu, we have detected up-regulation of its expression of approximately 2.5-fold (Figure 5(Fig. 5)) while no significant changes were observed after treatment with apigenin (Figure 5(Fig. 5)).

## Discussion

Bioactive potential of AP7Glu was analyzed and compared with apigenin. Obtained results demonstrated that sugar moiety in AP7Glu had important impact on biological activity of apigenin. 

Results on anti-candidal activity are in agreement with previous study by Mamadalieva et al. (2011[21]) who showed that AP7Glu is a more potent growth inhibitor of *Candida albicans *and* C. glabrata* when compared to apigenin. Both compounds had lower inhibitory potential but their fungicidal potential can cope with ketoconazole due to drugs primarily static effect. Observations reported herein, regarding the influence of apigenin and AP7Glu on the cytoplasmic membrane of *C. albicans,* are in accordance with recent studies showing that flavonoids localize either in the hydrophobic core of the lipid bilayer or at the membrane interface leading to corresponding alterations in the membrane (Selvaraj et al., 2015[27]). Ketoconazole, commercial antifungal drug that is widely used was shown not to have effect on membrane structure at tested concentration which is in accordance with previous studies (Uno et al., 1982[33]). Structures of the flavonoids as well as their ability to alter the membrane are both important factors that influence the nature and magnitude of their biological activity (Selvaraj et al., 2015[27]). AP7Glu caused more disturbances in membrane integrity upon treatment of *C. albicans* cells, which could be attributed to difference in chemical structure of the compounds.

ROS generation by *C. albicans* is dependent on morphogenesis and their highest levels are found in cells with hyphal form. Thus, ROS generation plays a major role in tissue invasion and infection (Sander et al., 2002[25]). Here we demonstrated decreasing amounts of extracellular and intracellular ROS in the presence of AP7Glu and apigenin which was shown to be dose-dependent. We propose that ROS inhibition by apigenin and AP7Glu could have important influence on the suppression of fungus pathogenesis, while ketoconazole does not have significant potential. AP7Glu was found to be more effective since it caused higher percentage of ROS inhibition that could subsequently lead to decreased pathogenesis. In addition, both compounds exhibited no influence on CYP51 enzyme inhibition which is the major target for commercial anticandidal drugs as ketoconazole. Unselective nature of ketoconazole CYP51 binding may be cause of known ketoconazole side effects (Lee et al., 2014[16]). By obtained results we propose that the major mechanism of anticandidal action of AP7Glu and apigenin is related to cell membrane disruption. *In vitro* inhibition of extra- and intracellular ROS in the cells of *C. albicans* indicated that both compounds may possibly lower the host tissue invasion and accordingly lead to decreased pathogenicity of *C. albicans*. In particular, AP7Glu showed higher potential for antimicrobial activity. It could inhibit growth of tested strains at lower concentration, cause membrane disturbance in shorter period of time and lead to lower levels of ROS in cells of *C. albicans,* which can contribute to lower tissue invasion by this fungi.

Beside anti-candidal potential we also analyzed cytotoxic effect of both compounds on HCT116 cells. Here we present that AP7Glu exhibited cytotoxic effect on colon cancer cells *in vitro*, as well as apigenin, by showing the ability to reduce cell's viability and induce cell death. Lee et al. (2014[16]) described that apigenin effectively inhibits viability of HCT116 cells and that treatment with apigenin results in chromosomal condensation and apoptotic bodies formation. We have shown for the first time that AP7Glu treatment of HCT116 cells was even more effective in reduction of cell's viability and induction of cell death. Induction of cell death was confirmed by flow cytometry showing that, after treatment with AP7Glu, HCT116 cells underwent significant necrosis. It has already been suggested that apigenin could induce apoptosis through p53-dependent pathway (Seo et al., 2012[28]) and that cells treated with apigenin exert elevated level of *p53* and *Bax *(Lu et al., 2010[18]). These results suggest that AP7Glu could also induce apoptosis through p53-dependent pathway. Moreover, our results showed that AP7Glu is even more potent in inducing apoptosis-associated *p53* and *Bax* genes expression in HCT116 cells. 

Interestingly, we have observed different effects of AP7Glu vs. apigenin on *SOX9* expression in HCT116 cells. Namely, AP7Glu led to increased expression of *SOX9* in HCT16 cells, while apigenin showed no significant effect. The elevated level of *SOX9* in derivative treated cells opened several questions. Some authors recognized SOX9 as unfavorable marker in patients with colorectal cancer, observing overexpression in 75 % of colorectal adenomas and 83 % of colorectal carcinomas (Lü et al., 2008[17]). Considering this fact, further upregulation of this gene could be an alarming sign of unfavorable effect. On the other hand, Bruun et al. (2014[2]) performed tissue microarray analysis of large consecutive, population-representative single-hospital series of primary colorectal carcinomas to explore the prognostic significance of SOX9 and could not find prognostic relevance. Therefore, further work is needed in order to understand the relevance and/or consequence of *SOX9* induction upon AP7Glu treatment.

The impact of flavonoids glycosylation on different bioactivities is still under intense investigation; being very complex and rather interesting issue and still requiring more data in order to enlighten general influence of sugar moieties in bioactivity assays. This is partly due to the lack of clinical and *in vivo* investigations that would support *in vitro* data. In some rarely available clinical trials *in vivo* data differ from that obtained *in vitro*. Some results indicated that glycosylated flavonoids, when applied *in vivo,* had similar or even better bioactivities (antidiabetic, antistress, antiallergic, antidegranulating, anti-inflammatory) compared to their respective aglycones (Xiao, 2017[36]). Recent findings demonstrated that apigenin glucosides appeared to enter cancer cells and are effectively hydrolyzed within the cells (Srivastava and Gupta, 2009[30]). 

As natural products, flavonoids are regarded as easily obtainable compounds, with promising role in cancer chemoprevention or agents in clinical antifungal treatment. Further study is needed to elucidate the precise mechanism of action in colon cancer model system for both apigenin and AP7Glu in order to apply their safe use. 

## Conclusion

This study highlighted AP7Glu, derivative of apigenin, as more biologically potent compound when compared with apigenin. Presented results indicated its antifungal activity comparable to the standard drug ketoconazole towards all *Candida* isolates, especially for fungicidal activity. Also, AP7Glu was more prominent in cytotoxic activity on colon cancer cells *in vitro *compared to apigenin*.*

## Notes

Marija Smiljkovic and Danijela Stanisavljevic equally contributed as first authors.

## Conflict of interest

The authors declare no conflict of interest.

## Acknowledgements

This work has been supported by the Serbian Ministry of Education, Science and Technological Development for financial support (Grant numbers 173032 and 173051). Binding studies to CaCYP51 has been supported by the Slovenian Research Agency (Grant P1-0010) and by programme of scientific and technological cooperation between the Republic of Serbia and the Republic of Slovenia „A combined methodology towards the development of novel, selective inhibitors of Candida CYP51“.

## Figures and Tables

**Table 1:**
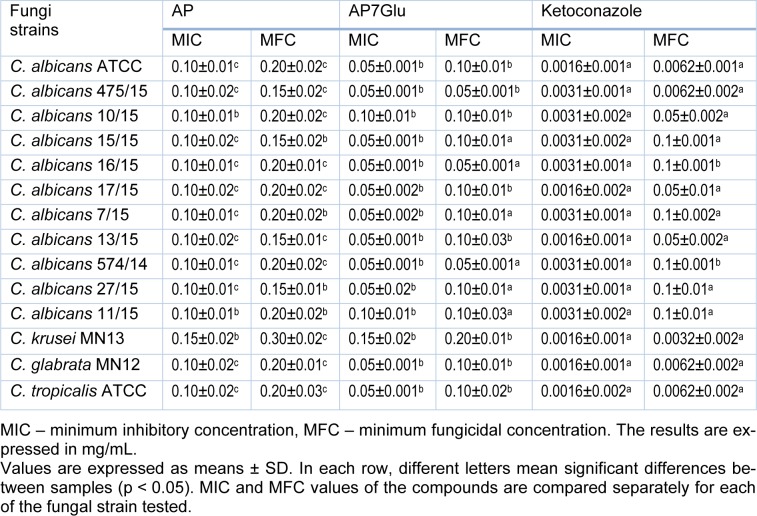
Activity of apigenin (AP), apigenin-7-*O*-glucoside (AP7Glu) and a reference compound ketoconazole against *Candida* strains in microdilution assay
